# Effect of MDP-Based Primers on the Luting Agent Bond to Y-TZP Ceramic and to Dentin

**DOI:** 10.1155/2018/2438145

**Published:** 2018-09-16

**Authors:** Sheila Butler, Bernie Linke, Ysidora Torrealba

**Affiliations:** ^1^School of Dentistry, Western University, London N6A 5C1, Canada; ^2^School of Dentistry, University of Alberta, Edmonton T6G 2R3, Canada

## Abstract

**Purpose:**

The aim of this study was to evaluate the influence of multimode MDP-based primers and different application protocols on the bond strength of a representative resin cement to an yttrium stabilized tetragonal zirconia (Y-TZP) ceramic.

**Materials and Methods:**

The occlusal dentin from 60 human molars was exposed. The teeth and zirconia cylinders (N = 60) (3 mm of diameter; 4 mm of height) were divided into six groups (n = 10) according to the ceramic surface conditioning: (1) air abraded with SiO_2_ particles; (2) Z-Prime Plus; (3) air abraded with SiO_2_ particles + Z-Prime Plus; (4) air abraded with SiO_2_ particles + All-Bond Universal; (5) air abraded with SiO_2_ particles + ScotchBond Universal Adhesive; and (6) untreated zirconia. The luting agent (Duo-Link cement) was applied on the treated dentin surface. Specimens were stored in water (37°C, 24 h) and tested in shear bond strength. Data were statistically analyzed using 2-way ANOVA and Post hoc Tukey tests (*α* = 0.05).

**Results:**

Significant effects of ceramic conditioning were found (p < 0.0001). The specimens sandblasted with silica particles followed by the application of Z-Prime Plus or All-Bond Universal presented greater bond strength values. For the untreated zirconia, several specimens failed prematurely prior to testing.

**Conclusions:**

Sandblasting with silica particles combined with Z-Prime Plus increased the bond strength.

## 1. Introduction

Yttrium stabilized tetragonal zirconia polycrystals (Y-TZP) ceramic is a material used for the restoration of teeth with primarily full coverage restorations. The zirconia crowns can be milled monolithically to full contour or alternatively the core of zirconia can be milled followed by layering of feldspathic porcelain onto the core. The layering of the porcelain onto the core of the zirconia has resulted in superior esthetics along with issues of chipping of the veneered porcelain [1–3].

In some situations, the remaining tooth structure for a full coverage crown does not allow for adequate retention/resistance form. Therefore, the options are either increasing the clinical crown height with crown-lengthening procedures to facilitate restorative therapy [4, 5] or increasing the bond of the cement to the remaining tooth structure [6] and the zirconia [7].

Zirconia does not contain an amorphous silica glass and as a result does not allow for traditional ceramic surface treatment [8, 9]. Various surface treatments, both mechanical and chemical, of zirconia have been attempted with varying results. These treatments include ultrasonic cleaning, abrasion, sandblasting, hydrofluoric acid etching, silanization, and application of surface bonding adhesives [10, 11].

One of the ongoing concerns with increasing the internal surface area for bonding by mechanically altering the surface of zirconia with a diamond bur or sandblasting is the development of a phase change in the zirconia and as a result introducing flaws into zirconia microstructure [12–14]. The resulting reduction in physical properties of the zirconia could result in a weaker restoration and compromising the long-term prognosis. Recent research would indicate that this may not be the case and under certain sandblasting conditions may actually increase the zirconia strength due to transformational toughening, along with providing some surface roughness for micromechanical retention possibilities for cement of zirconia [5, 14, 15].

The use of MDP (10-methacryloyloxydecyl dihydrogen phosphate) as a multimode functional adhesive monomer has been postulated to provide increased retention for cements used in the cementing of zirconia crowns by increasing the bond to zirconia as well as to dentin [10, 11]. It has been proposed that the methacryloxy end of the phosphate compound couples with the resin matrix, and phosphoric acid group react with the zirconia surface. The ability of surface treatments to improve the retention of cements to zirconia has had varying results.

The aim of this study is to evaluate the influence of various multimode MDP-based primers and different application protocols on the bond strength of a representative resin cement to a yttrium stabilized tetragonal zirconia (Y-TZP) ceramic as well as of zirconia to dentin.

## 2. Materials and Methods

Partially sintered zirconia disks (NexxZr, Sagemax Bioceramic, Berlin, Germany, Shade: WT, LOT: HFMCG) were used to obtain zirconia cylinders of 4 mm in height and 3 mm in diameter using a computer-aided manufacturing technique (vhf CAM S1 Impression Milling Machine, Ammerbuch, Germany). Sixty zirconia cylinders were machined under water coolant (IsoMet® 1000, Buehler, Lake Bluff, IL, USA). The cylinders were subsequently sintered in a VITA Zyrcomat 6000 MP furnace (VITA, Bad Säckingen, Germany), according to the manufacturer's instructions. All specimens were polished using Diamond Lapping Films (Allied High Tech Products Inc, Compton, CA, USA) in a decreasing sequence of abrasive size from 30, 15, 9 to 3 *μ*m, under copious water cooling.

Sixty noncarious human molars extracted for periodontal or orthodontic reasons were collected under ethical approval from the University of Alberta Ethics Committee In Human Research (Pro00063336). The teeth were scaled, cleaned, stored in 0.5% chloramine solution at 4°C to prevent bacterial growth, and used within 3 months following extraction. The crowns were sectioned horizontally with a low speed and intense water cooling to remove the occlusal morphology. The teeth were then ground perpendicularly to their longitudinal axes using P320 SiC paper (Schmitz Metallographie GmbH, Herzogenrath, Germany) until all occlusal enamel was completely removed [16, 17]. The teeth were embedded in acrylic resin (Orthodontic Resin, Dentsply) up to 2.0 mm below the cemento-enamel junction. The ground dentin surface was then cleaned using a rubber cup and fine pumice water slurry. Subsequently a smear layer was created by wet-grinding the dentin surface with P600 SiC paper for 60 s. The prepared coronal dentin was treated as follows: lightly air-dried in an oil free air-stream and then etched with 37% phosphoric acid for 15 s and washed with 10 mL of distilled water and the excess water was removed with absorbent paper. Two layers of adhesive (All-Bond Universal, Bisco, Schaumburg, IL, USA, LOT: 1600004095) were applied to the coronal dentin. For each layer any excess adhesive was removed with a dry microbrush, prior to gentle air-drying. The adhesive was light cured for 10 s perpendicular to the dentine surface from a distance of 2 mm using a Mini LED (Satelec Acteon) with an irradiance of 1250 mW/cm^2^. The prepared teeth were finally randomly assigned to six groups of ten teeth.

Prior to surface conditioning, all ceramic blocks were ultrasonically cleaned (Bransonic® ultrasonic cleaner, Branson Ultrasonic, Danbury, CT, USA) for 5 minutes in distilled water. The ceramic cylinders were randomly divided into six groups (n = 10) with each group receiving a different conditioning treatment to the bonding surface. Group (1) was air abraded with SiO_2_ particles; (2) primed with a thin layer of Z-Prime Plus (Bisco, Schaumburg, IL, USA, LOT: 1600004092); (3) air abraded with SiO_2_ particles and primed with a thin layer of Z-Prime Plus; (4) air abraded with SiO_2_ particles and coated with a thin layer of All-Bond Universal; (5) air abraded with SiO_2_ particles and coated with a thin film of ScotchBond Universal Adhesive (3M ESPE, Seefeld, Germany, LOT: 632027). A final group of ten specimens (6) were left as the untreated zirconia control.

Particle air abrasion was performed by making circular movements with the nozzle of the air abrasion unit at a distance of 10 mm, with 2.8-bar pressure for 15 s using 30 *μ*m diameter SiO_2_ particles (Rocatec™ Soft, 3M ESPE, Seefeld, Germany, LOT: 450384).

A small portion of Duo-link cement (Bisco, Schaumburg, IL, USA, LOT: 1600003948) was placed on the treated ceramic surface. The zirconia cylinder was placed on the treated dentin surface using a positioning device that allowed for the application of a consistent 750 g load on the ceramic-resin cement-dentin block. Excess cement was carefully removed using a microbrush and the resin cement light cured (Mini LED Satelec Acteon, 1250 mW/cm^2^) for 10s each from two opposite sides of the ceramic cylinder. After ten minutes following resin cementation, the specimens were transferred to storage in distilled water for 24 h at 37°C prior to shear bond strength determination.

Shear bond testing was performed in a universal testing machine (ElectroPuls 3000, Instron GmbH, Darmstadt, Germany) with loading applied in a knife-edge configuration using a crosshead speed of 0.5 mm/min. Each specimen was rigidly positioned to ensure that the adhesive interface was parallel and as close as possible to the knife displacement axis. The shear bond strength (SBS) (MPa) was calculated from the recorded failure load (*P*) and the cross-sectional area of the adhesive interface where

(1) $$\begin{array}{c} SBS=\frac{P}{\pi r^{2}} \end{array}$$

and *r*, the radius of the bonding interface, was 1.5 mm.

The fractured surfaces of all the tested specimens were visualized in a stereo microscope at up to 50x magnification (Leica M125, Wetzlar, Germany) to discriminate the failure mode. Further specimens with representative fractures were selected for imaging using scanning electron microscopy (SEM). Specimens were mounted on a metallic stub, sputter coated with 7 nm of platinum in a Polaron E5100 coating unit (Polaron Equipment, Ltd., Bedford, UK), and observed at x20 and x500 magnifications (Hitachi S-2500, Hitachi, Mito City, Japan).

The shear bond strength (MPa) data were statistically analyzed using a two-way analysis of variance (ANOVA) where the factors were surface finish (at two levels) and the use of a surface primer/adhesive (at four levels) followed by post hoc Tukey tests (*α* = 0.05). P values less than 0.05 were considered to be statistically significant in all tests. Specimens that failed prematurely during the aging conditions were considered as 0 MPa for statistical analysis.

## 3. Results

**Figure 1:**
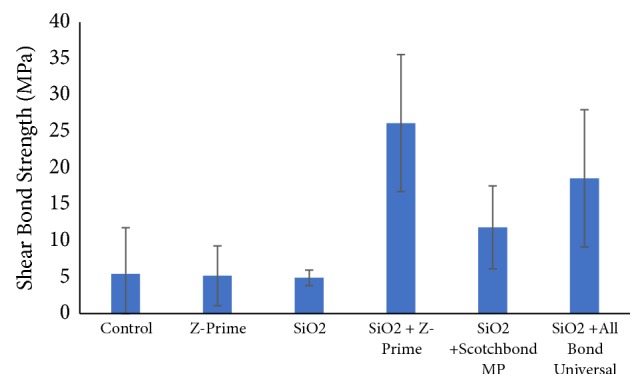
The mean bond strength values (MPa) according to the surface treatment.

A two-way ANOVA demonstrated a significant impact of modifying the zirconia surface with SiO_2_ particle air abrasion prior to cementation on the recorded shear bond strength (SBS) (p < 0.001). Furthermore, the SBS was significantly influenced by the use of a surface primer/adhesive but the pattern of change in SBS was associated dependent on zirconia surface condition (p < 0.001). Post hoc Tukey tests demonstrated that the use of Z-Prime Plus and All-Bond Universal to condition the SiO_2_ particle air abraded zirconia surface, resulted in significantly higher bond strengths (p < 0.001) than the corresponding unconditioned surface (Table 1). The mean bond strength values and associated standard deviations for each group are shown in Table 2 and Figure 1. No surface modification, SiO_2_ particle air abrasion only, and use of Z-Prime Plus only promoted low and unstable bond strengths with several pretest failures recorded. In contrast, air abrasion with silica particles resulted in higher and more consistent bond strengths (less pretest failures) when combined with Z-Prime Plus and also All-Bond Universal. Air abrasion with silica particles in combination with Z-Prime Plus or All-Bond Universal allowed the highest mean SBS values.

**Figure 2:**
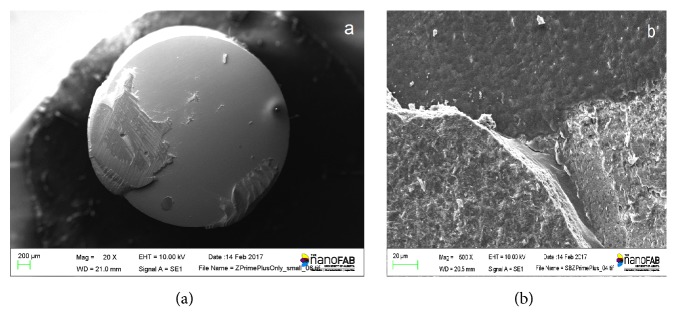
Representative micrographs of the debonded surfaces: (a) mixed failure – residue of cement left on the left side of the debonded surface from Z-Prime Plus only group; (b) mixed failure exhibiting dentin tubules and cement from SB + Z-Prime Plus group.

A summary of failure types is presented in Table 3 and representative micrographs of the debonded surfaces are exhibited in Figure 1. Adhesive failures (between the luting cement and Y-TZP surface) took place for the majority of specimens. No adhesive failures involving the cement-dentin surface were observed. In addition, mixed failures were observed as shown in Figure 2.

## 4. Discussion

This study evaluated the influence on bond strength of multimode MDP-based primers and application protocols on the bond strength of resin cement to dentin and a yttrium stabilized tetragonal zirconia polycrystal (Y-TZP) ceramic.

The shear bond strength test (SBS) was used to evaluate the adhesive interface between dentin, resin cement, surface treatment modalities, and the Y-TZP. Shear bond strength tests evaluate the cohesive strength of the base material better than the bond strength of the adhesive interface [18]. The concern with a SBS is that the test increasingly stresses the base components with possible deformation of those substrates and this flexing may actually test the resistance of the components to bending forces of the substrates and not the adhesive complex. The examination of the fractured surfaces in this current study exhibited the predominance of adhesive failures between ceramic and cement as shown in Table 3.

There is a concern with air abrasion to Y-TZP because this process may induce stresses that result in crack propagation [19–21]. Recently a study using 4-point flexural test indicated that if 30 micron Si-coated Aluminum oxide was used at 10 mm with 2.8-bar pressure for 15 seconds then there would be no reduction in flexural strength. In this study, 30 micron SiO_2_ was used [14].

Significant effects of ceramic conditioning were found (p < 0.0001). The specimens air abraded with SiO_2_ particles followed by the application of Z-Prime Plus or All-Bond Universal resulted in higher bond strength values. For the untreated zirconia, several specimens failed prematurely prior to testing which is attributed to the unstable and weak bond strength present in this group.

The results of this study would indicate that air abrasion with 30 *μ*m of SiO_2_ particle with no further surface treatment does not improve the bond strength. However, if subsequent to air abrasion a multimode MDP application is completed to Y-TZP, there is a significant difference in the bond strength. There have been various explanations provided for this fact, including (a) mechanical surface abrasion and cleaning of the surface, (b) silica deposition, (c) increased chemical reactivity energy levels, or (d) a combination of all the preceding reasons [22].

There are differences among the studies regarding sandblasting consequences likely due to the varying size of particles, pressure, distance, and angle of application. Nevertheless, surface roughness alone would not account for improved bond strength as there was no significant difference in bond strength between untreated zirconia and the specimens which were only sandblasted. Additionally, this study demonstrated that without air abrasion the application of a multimode MDP adhesive monomer did not provide an increase in bonding strength.

Chen in 2011 [22] has shown that sandblasting with Si-coated alumina particles results in a surface layer of SiO_2_ deposited on the zirconia surface resulting in increased reactivity between that layer and a MDP adhesive monomer such as All-Bond Universal and ScotchBond Universal. The phosphate ester monomers from MDP have been shown to react with Y-TZP [23].

Z-Prime is a zirconia primer that contains a mixture of organophosphate and carboxylate monomers. The organophoshate monomer has an organofunctional end that can polymerize with the resin cement [13]. The synergistic coupling of phosphate and carboxyl functional monomers can bond to the metal oxides with the Y-TZP resulting in an increase in the bond. The findings of this study would confirm that observations as the highest values were obtained by SB + Z-Prime Plus. There was no significant difference between SB + Z-Prime Plus and SB + All-Bond Universal. All-Bond Universal and ScotchBond Universal are a multimode MDP primer. Although the bonding values were higher for SB + ScotchBond Universal than untreated zirconia, there was no significant difference between these two groups. This could be a result of the slightly lower pH value provided by the ScotchBond Universal resulting in decreased copolymerization of the resin cement used in this study.

Evaluation of the mode of failure indicated that no complete failures occurred at the dentin- cement interface and this is consistent with the findings of other studies [24]. This indicated the bond to dentin is greater than the bond to zirconia.

Failures for SB + Z-Prime Plus took place mainly at the Y-TZP and cement interface. Given that all samples failed in the untreated zirconia at this interface this is not an unexpected finding and would indicate that given zirconia's dense tetragonal structure there is minimal mechanical retention provided by the material itself. When the zirconia was sandblasted there were less failures at the cement-zirconia interface indicating some change has occurred to the Y-TZP surface. The addition of Z-Prime Plus to the untreated surface changed the mode of failure in some samples but did not increase the bond strength for that of the untreated zirconia. When the samples were sandblasted and then conditioned with Z-Prime Plus, All-Bond Universal, or ScotchBond Universal, changes occurred to the mode of failure compared to the untreated zirconia. This factor indicates a positive effect on bond strength at the Y-TZP-cement interface.

## 5. Conclusion

Within the limitations of this study, Y-TZP surface sandblasted with 30 micron SiO_2_ particles prior to conditioning the bonding surface significantly increased the bonding of Y-TZP to dentin. The SB + Z-Prime Plus protocol resulted in the highest bonding values.

## 6. Clinical Relevance

Zirconia surface air abraded with SiO_2_ particles and primed with a thin layer of Z-Prime Plus is the recommended surface treatment before cementing a zirconia-based restoration.

## Figures and Tables

**Table 1:** Two-way ANOVA results of the shear bond strength (SBS) data.

**Source**	**DF**	**SS**	**MS**	**F value**	***p* value** ^**∗**^
Corrected Model	5	3829	766	17.2	0.001
Factor 1: Surface Finish	1	1043	1043	23.4	0.001
Factor 2: Conditioning Primer	3	1327	442	9.9	0.001
Factorial Interaction	1	1157	1157	25.9	0.001
Corrected Total	59	6240			

^*∗*^p < 0.05.

**Table 2:** Mean values (MPa) and standard deviations (in parentheses) of the shear bond strength obtained for the different ceramic surface conditioning.

**Untreated zirconia**	**SB only**	**Z-Prime Plus only**	**SB** + **Z-Prime Plus**	**SB** + **All-Bond Universal**	**SB** + **ScotchBond Universal Adhesive**
5.45	4.91	5.18	26.15	18.56	11.83
(6.34)^a^	(1.07)^a^	(4.11)^a^	(9.41)^b^	(9.42)^b,c^	(5.69)^a,c^

Identical letters indicate no statistically significant differences (*P* > .05).

**Table 3:** Number of specimens per group (n), number of pretest failures (PTF), and failure types of the debonded specimens. Failure between ceramic and cement (Adhes-C-cem); failure between dentin and cement (Adhes-D-cem); cohesive failure of cement and ceramic (MIX); cohesive failure of the ceramic (C-cer); cohesive failure of the cement (C-cem).

**Study factors**	**n**	**PTF**	**FAILURE TYPES**				
**Ceramic conditioning**			**Adhes-C-cem**	**Adhes-D-cem**	**C-cer**	**C-cem**	**MIX**
**Untreated zirconia**	10	4	10	0	0	0	0
**SB only**	10	0	6	0	0	3	1
**Z-Prime Plus only**	10	1	7	0	0	0	3
**SB + Z-Prime Plus**	10	0	8	0	0	2	0
**SB + All-Bond Universal**	10	0	3	0	0	1	6
**SB + ScotchBond Universal Adhesive**	10	0	5	0	0	3	2

## Data Availability

The data used to support the findings of this study are available from the corresponding author upon request.
